# Mitotic Kinases Aurora-A, Plk1, and Cdk1 Interact with Elk-1 Transcription Factor through the N-Terminal Domain

**DOI:** 10.1155/2024/6798897

**Published:** 2024-04-30

**Authors:** Oya Arı Uyar, Yigit Koray Babal, Bayram Yılmaz, Isil Aksan Kurnaz

**Affiliations:** ^1^Yeditepe University, Biotechnology Graduate Program, 26 Agustos Yerlesimi, Kayisdagi, 34755 Istanbul, Türkiye; ^2^Molecular Neurobiology Laboratory (AxanLab), Department of Molecular Biology and Genetics, Gebze Technical University, 41400 Gebze, Kocaeli, Türkiye; ^3^Institute of Biotechnology, Gebze Technical University, 41400 Gebze, Kocaeli, Türkiye; ^4^Yeditepe University, Faculty of Medicine, Department of Physiology, 26 Agustos Yerlesimi, Kayisdagi, 34755 Istanbul, Türkiye

## Abstract

Elk-1 is a member of the ETS domain transcription factor superfamily that is phosphorylated upon mitogen-activated protein kinase (MAPK) pathway activation, which in turn regulated its interaction with partner protein serum response factor (SRF), leading to formation of a ternary complex with DNA. It has previously been reported that Elk-1 interacts with a mitotic kinase Aurora-A, although the mechanisms or the relevance of this interaction was unclear. Elk-1 was also reported to be phosphorylated by CDK5 on Thr417 residue. In this study, we show for the first time that this transcription factor interacts not only with Aurora-A but also with other mitotic kinases Aurora-B, Plk1, and Cdk1, and we define the interaction domain on Elk-1 to the first N-terminal 205 amino acids. We also describe putative phosphorylation sites of these mitotic kinases on Elk-1 and show that Elk-1 peptides containing these residues get phosphorylated by the mitotic kinases in *in vitro* kinase assays. We also perform bioinformatic analysis of mitotic phosphoproteomes and determine potential interaction partners for Elk-1 in Plk or Aurora phosphoproteomes. We propose that understanding the dynamic phosphorylation of Elk-1 by mitotic kinases is important and that it can present a novel target for anticancer strategies.

## 1. Introduction

The ternary complex factor (TCF) subfamily of the ETS domain transcription factor superfamily includes Elk-1, which is phosphorylated by mitogen-activated protein kinase (MAPK) signaling pathway on serines 383 and 389, which in turn leads to formation of a ternary complex of Elk-1/SRF/DNA [1–4]. Elk-1 was previously shown to be phosphorylated not only by ERK/MAPKs but also by protein kinase C (PKC) and phosphatidyl inositide-3 kinase (PI3K) pathways [5–7]. Phosphorylation of Elk-1 on threonine 417 was shown to correlate with differentiation grade of colonic adenocarcinomas, and this phosphorylation was reported to be mediated by cyclin-dependent kinase CDK5 [8, 9].

Our laboratory had previously shown that Elk-1 interacted with microtubules and dynein motor protein in neurons, as well as mitotic spindle in dividing brain tumor cells; we further showed Elk-1 to colocalize and interact with mitotic kinase Aurora-A (Aur-A) [10–12]. Aurora kinases (Aur-A, Aur-B, and Aur-C) are mitotic kinases that regulate centrosome separation, maturation, and cytokinesis [13, 14]. Aur-A and other mitotic kinases were recently identified among the key culprits behind glioblastoma (GBM) recurrence and thus are under scrutiny for the development of anticancer treatments [13, 15–19]. It is therefore intriguing that Elk-1 was identified as one of the 572 validated targets that result in mitotic defects when absent, in a large-scale genome-wide phenotypic profiling of 21,000 human genes for mitotic effects using siRNA knockdown [20]. Therefore, in this study, we wanted to further study the interaction of Elk-1 protein with selected mitotic kinases, namely, Aur-A, Aur-B, Plk1, and Cdk1, for which putative phosphorylation motifs were identified. We identified that Elk-1 can indeed interact with all four mitotic kinases, and we narrowed down the interaction domain to be in the first 205 amino acids on the N terminus of Elk-1 protein. We further identified potential interaction and phosphorylation motifs for each mitotic kinase and showed that in an *in vitro* kinase assay, all four mitotic kinases were capable of phosphorylating an Elk-1 peptide.

It is important to note that in a mitotic phosphoproteome analysis, one of the putative phosphorylation sites, S304, was found to be phosphorylated and identified as a mitotic substrate of Polo-like kinase 1 (Plk1) [21]. A similar study mapped Elk-1-Ser304-Ser324 phosphorylations in Plk and Aur phosphoproteomes [22], and a quantitative mitotic phosphoproteome analysis confirmed Elk-1 Ser 304, Ser324, and Ser326 phosphorylations to be specific for mitosis [23], validating the physiological relevance of our biochemical studies. Analyzing these mitotic phosphoproteome datasets further, we show that Elk-1 interacts with proteins such as KLF4 in PLK phosphoproteome, with proteins such as SRF, CEPT1 in Aur/PLK phosphoproteome, and with emerin (EMD) protein in both phosphoproteome datasets. These proteins are involved in mitosis either through regulation of metabolism, G1/S transition, or actin-tubulin dynamics, indicating a potential novel role for Elk-1 in mitosis in tumor cells.

## 2. Materials and Methods

### 2.1. Plasmids

Either empty pGEX-4T2 plasmid or pGEX-4T2-Elk-1 (1-93), pGEX-4T2-Elk-1 (1-205), pGEX-4T2-Elk-1 (205-428), and pGEX-4T2-Elk-1 (349-428) carrying various Elk-1 deletions fused to GST were a gift of Prof. A.D. Sharrocks.

### 2.2. Cell Culture

Glioblastoma cell lines U138 (ATCC HTB-16) and U87 (ATCC HTB-14, glioblastoma of unknown origin) and neuroblastoma cell line SH-SY5Y (ATCC CRL-2266) were grown in DMEM contains 4.5 g/l glucose, 10% FBS, 1× penicillin/streptomycin, and 1× L-glutamine (these cells do not appear in ICLAC database of cross-contaminated or misidentified cell lines). For transient transfections, cells were seeded into 10 cm tissue culture dishes at 1.5 × 10^6^ cells or into 6 well plates at 3 × 10^5^ cells, as indicated, and incubated for 24 hours [10, 24]. Transfection mixtures were prepared (10 *μ*g DNA and 30 *μ*g PEI reagent (Polysciences 23966-1) for 1.5 × 10^6^ cells and 2 *μ*g DNA and 5 *μ*g DNA for 3 × 10^5^ cells) to obtain 1 : 3 ratio, vortexed, and incubated for 15 min at room temperature. Cells were then incubated for 48 hr.

### 2.3. Western Blot

Essentially, cell lysates were prepared in RIPA cell lysis buffer containing protease and phosphatase inhibitor cocktails (Roche); samples were resolved in 10% SDS-PAGE gel and transferred to a nitrocellulose membrane (Millipore). The membrane was blocked for 1 hr in 3% BSA solution at room temperature, followed by incubation with primary antibodies overnight at 4°C at indicated dilutions (mouse monoclonal Aurora A (BD Biosciences 610938) 1 : 1000, Aurora B (BD Biosciences 611082) 1 : 1000, Plk1 (Abcam ab17056) 1 : 2000, Cdk1 (Abcam ab32384) 1 : 5000, rabbit polyclonal total Elk-1 (Cell Signaling #9182) 1 : 1000, rabbit P-S383-Elk-1 (Cell Signaling #9181) 1 : 1000, rabbit p44/42 MAPK (Erk1/2) (Cell Signaling #9102) 1 : 1000, rabbit SRF (Santa Cruz Biotechnology sc-335) 1 : 1000, and rabbit GST (Cell Signaling #2622) 1 : 1000). Blots were then washed 3 times in TBS-T and incubated with secondary antibodies (goat anti-mouse IgG-HRP, Santa Cruz sc-2005 or anti-rabbit IgG- HRP, Santa Cruz sc-2004) at 1 : 5000 dilution in BSA for 1 hr. After 3 washes in TBS-T, signal was developed with Immobilon Crescendo Western HRP substrate (Millipore) and captured with either Bio-Rad or DNS chemiluminescent imaging systems.

### 2.4. Immunoprecipitation

U87 or SH-SY5Y cells were subcultured into 10 cm dishes at 1.5 × 10^6^ cells/dish. For mitotic arrest and IP, U87 cells were blocked at prometaphase by incubating with 100 ng/ml nocodazole in growth medium for 16 hr and released by incubating in normal growth medium; thereafter, cells were collected at 30 min intervals by mitotic shake-off. For IP from whole cell lysates, U87 or SH-SY5Y cells were collected 48 hr after transfection, or for IP of endogenous proteins 72 hr after plating. Collected cells were washed with ice-cold PBS and lysed in RIPA buffer (CST, #9806). Lysates were incubated with 2 *μ*g rabbit polyclonal anti-Elk-1 antibody (Santa Cruz Biotechnology sc-365876) overnight at 4°C; antibody-protein complexes were precipitated with protein A agarose beads (Sigma P2545) and washed 1× with lysis buffer and 2× with PBS. Proteins were then denatured in SDS-loading buffer and analyzed by Western blotting as described above.

### 2.5. GST Pulldown Assay

BL21(DE) pLysS bacterial stock was thawed, and competent cells were prepared by CaCl_2_ treatment. Either empty pGEX-4T2 plasmid or the carrying Elk-1 deletion GST fusions (obtained from Prof. A.D. Sharrocks) were transformed into BL21(DE) pLysS in the presence of 33 *μ*g/ml chloramphenicol and 50-200 *μ*g/ml ampicillin. One colony from each transformation was inoculated into 10 ml LB medium with ampicillin and chloramphenicol for overnight at 37°C at 200 rpm. Next day, 3 ml of overnight culture was inoculated in 300 ml of LB broth containing ampicillin and chloramphenicol and grown until OD_600_ 0.5-0.7. 0.5 mM isopropyl *β*-D-1-thiogalactopyranoside (IPTG) was used to induce protein expression at 37°C for 4 hr; bacteria were collected by centrifugation at 5000 rpm for 10 minutes. Pellets were resuspended in ice-cold PBS containing 1 : 1000 protease inhibitor cocktail, 1 mM sodium orthovanadate and 1 mM PMSF as phosphatase inhibitors, %1 Triton 100×, and 1 mM DTT. Bacteria were lysed by sonication, and protein lysate was cleared by centrifugation at 4°C and 15,000 rpm for 15 minutes. Bacterial protein expression was confirmed by SDS-PAGE and Coomassie staining. For pulldown experiments, GST-Elk-1 variants were semipurified using glutathione-sepharose beads (Sigma G4510) as per the manufacturer's instructions, followed by incubation with U87 glioblastoma cell lysates. Samples were then analyzed with Western blot using primary antibodies specific for Aur-A, Aur-B, Cdk1, Plk1, ERK, and SRF as described above; GST-Elk-1 deletion “input” samples were analyzed with GST antibody in Western blots.

### 2.6. *In Vitro* Protein Kinase Assay

The kinase reaction was set up with the incubation of Elk-1 protein with specific kinases. 0.1 *μ*g Elk-1 protein (CST #9183) was incubated with either Aurora A (Millipore 14-511, active, recombinant N-terminal His-tagged), Aurora B (CST #7394, active recombinant protein), Cdk1/Cyclin B1 (Millipore 14-450; active C-terminal His-tagged full-length Cdk1 and GST-tagged human full-length cyclin B), or Plk1 (CST #7728) active kinases (0.2 ug or 0.5 ug kinase, see Results for details) at 37°C for 1 hour, as per manufacturer's instruction (Universal Kinase Assay Kit, Abcam ab138879). Recombinant hexokinase (R&D Systems cat. no. 8179-HK-020) was used as negative control (generous gift of Prof. Dr. Nuri Ozturk, GTU). Then, 20 *μ*l kinase reaction was combined with 20 *μ*l ADP sensor buffer and 10 *μ*l ADP sensor composed of the mixture of ADP sensors I and II. The mixture was incubated in the dark for 15 minutes, and the fluorescence intensity was measured by spectrophotometry at 540 nm excitation and 590 nm emission.

### 2.7. *In Silico* Analysis of Predicted Phosphorylation Motifs

Mouse Elk-1 protein sequence (NCBI ID NM_007922) was analyzed for potential phosphorylation motifs through a range of different online prediction tools:

http://ppsp.biocuckoo.org/

http://kinasephos2.mbc.nctu.edu.tw

http://phosphosite.org

The unified prediction was formed, based on phosphothreonine and phosphoserine motifs and/or search of the protein sequence for consensus motifs for selected kinases (Aur-A and Aur-B, Plk1, and Cdk1).

### 2.8. Protein-Protein Interaction Network Construction from Elk-1 Transcriptome and Phosphoproteome Integration

Elk-1 specific protein-protein interaction (PPI) network was constructed with the integration of human whole protein-protein integration network from BioGRID [25] and Elk-1 overexpression microarray dataset reported elsewhere [26] using KeyPathwayMiner algorithm [27]. Binarized differentially expressed genes from microarray dataset (significant genes *p* value < 0.05 assigned as 1 and *p* value > 0.05 as 0) and human whole PPI network were used as an input for KeyPathwayMiner using INES strategy and GREEDY algorithm with node exception 3 parameter. The Elk-1-specific PPI network was filtered with Plk-Aur phosphoproteome [22] and Plk phosphoproteome [21], as well as mitotic phosphoproteome [23] datasets. Then, Elk-11 and interaction partners were filtered to construct the subnetwork. All networks were visualized by Cytoscape software [28].

## 3. Results

### 3.1. Putative Mitotic Kinase Phosphorylation Motifs and Interaction with Mitotic Kinases

**Figure 1:**
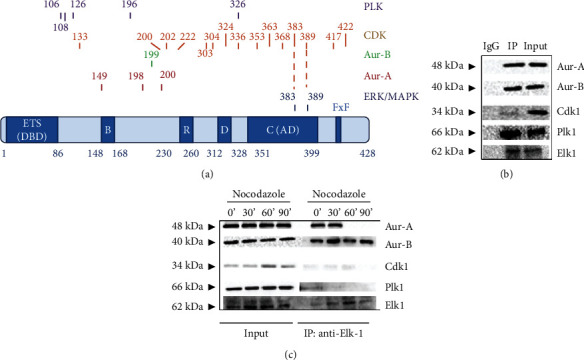
Interaction of Elk-1 with mitotic kinases. (a) A schematic diagram of the domain structure and predicted mitotic kinase phosphorylation motifs of ternary complex factor Elk-1; predicted Plk phosphorylation motifs are shown in purple, Cdk motifs in orange, Aur-B motif in green, Aur-A motifs in red, and the known ERK/MAPK phosphorylation sites are shown in blue; ETS: E-twenty-six; DBD: DNA binding domain; B: SRF interaction domain; R: repression domain; D: docking domain; C: activation domain (AD). (b) Immunoprecipitation of endogenous Elk-1 in SH-SY5Y cell lines, followed by Western blot with antibodies specific for Aur-A, Aur-B, Cdk1, Plk1, and Elk-1 (IgG is used as IP control). (c) U87 cells were arrested with nocodazole treatment, released into mitosis at time 0 (indicated as 0′), and samples were taken at 30 min (30′), 60 min (60′), and 90 min (90′) after release. Immunoprecipitation was performed using Elk-1 antibody, and both input and IP samples were analyzed by Western blot with antibodies specific for Aurora-A, Aurora-B, Cdk1, Plk1, and Elk-1.

To address the question of whether there are any phosphorylation motifs for mitotic kinases on Elk-1, we have performed an *in silico* prediction of putative phosphorylation sites (see Materials and Methods) and identified several putative sites for Plk (Ser106, Thr108, 126, 196, and Ser326), Cdks (Thr133, Ser200, Ser202, 222, Ser303, Ser304, Ser324, 336, 353, 363, 368, Ser383, Ser389, Thr417, and 422), Aur-A and Aur-B (Ser149, Ser198, Thr199, and Ser200), in addition to the well-known MAPK sites (Ser383, Ser389) (Figure 1(a)) [4].

Elk-1 harbors the consensus ETS DNA binding domain (residues 1-86) overlapping with nuclear localization signal (NLS) and the SRF interacting B domain (residues 148-168) [1, 2]. In the central segment of the protein, there is the repression (R) domain (residues 230-260), where SUMOylation of K230, K249, and K254 was shown to be critical to the repression activity [1, 4]. The docking domain (D, residues 312-328) was identified as the ERK/MAPK docking region of Elk-1, along with the FxF motif [3], and the transactivation domain (TAD, or C domain, residues 351-399) was shown to be critical for the transactivation function upon phosphorylation of Ser383 and Ser389 by ERK/MAPK (Figure 1(a)) [3, 4].

Since colocalization and interaction of Elk-1 with Aur-A kinase were previously shown in U87 cell lines [12], we next asked whether Elk-1 also interacts with Aur-B, Cdk1, or Plk1 in a different cell line to confirm that the interaction is independent of cell context. To that end, we first immunoprecipitated (IP'ed) endogenous Elk-1 from SH-SY5Y neuroblastoma cells and showed that it interacts with all four mitotic kinases—Aur-A, Aur-B, Plk1, and Cdk1—albeit to a lower extent in the case of Cdk1; however, no interaction was observed when a control IgG was used in immunoprecipitation (IP lane vs. IgG lane; Figure 1(b)).

To analyze whether this interaction is mitosis dependent, we arrested the highly proliferating U87 cells with nocodazole, and lysates were collected 0, 30, 60, and 90 min after release from mitotic arrest. In accordance with previous reports, Aur-A was confirmed to interact with Elk-1 only at time 0 and 30 min, but not at 60 or 90 min; however, Aur-B interaction was present at all time points (Figure 1(c)). Some Plk interaction was visible at the onset of mitosis (0 min), whereas very low level of Cdk interaction was found at all time points (Figure 1(c)). Reverse IP with either wild-type, kinase-active (T161E), or kinase-dead (T161A) cotransfected Cdk1 plasmid showed Elk-1 interaction (Supplementary File, slides 16-19).

### 3.2. Identification of the Region of Interaction on Elk-1 and the Effect of Phosphomutants on Elk-1/Mitotic Kinase Interactions

**Figure 2:**
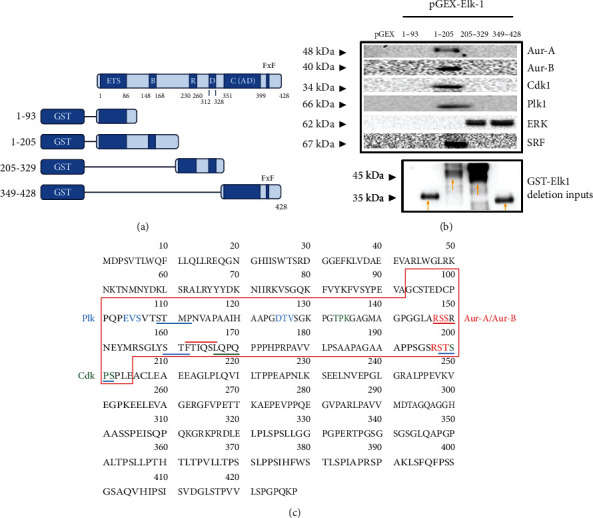
Interaction of mitotic kinases with Elk-1 protein. (a–c) Domain of interaction on Elk-1 protein: (a) a schematic diagram of GST-Elk-1 deletion mutants in pGEX-2T vector used in this study; (b) GST pulldown analysis of Elk-1 and mitotic kinase interaction; GST alone (pGEX) or GST-Elk-1 deletions were expressed in BL21 pLysS strain and semipurified using glutathione-sepharose beads, followed by incubation with U87 glioblastoma cell lysates; pulldown samples were analyzed with Western blot using primary antibodies specific for Aur-A, Aur-B, Cdk1, Plk1, ERK, and SRF; GST-Elk-1 deletion inputs were analyzed with GST antibody (lower panel); (c) putative binding motifs (blue line for Plk1, green line for Cdk1, and red line for Aurora-A or Aurora-B) on Elk-1 protein sequence for the mitotic kinases and their predicted phosphorylation sites (blue font for Plk1, green font for Cdk1, and red font for Aurora-A or Aurora-B) within amino acids 93-205 (red box).

To analyze the domain of interaction for mitotic kinases, we have expressed a series of Elk-1 deletion constructs as GST fusions, GST-Elk-1 (1-93), GST-Elk-1 (1-205), GST-Elk-1 (205-329), and GST-Elk-1 (349-328) (Figure 2(a)) and carried out pulldown assays. Since immunoprecipitations have been performed with SH-SY5Y cells, we have performed the GST pulldown assays using U87 cell lysates. When samples were analyzed for endogenous Aurora-A, Aurora-B, Cdk1, and Plk1, interaction was only present in GST-Elk-1 (1-205) region, while no pulldown was observed in pGEX alone, or in other GST-Elk-1 deletions (Figure 2(b)). Pulldown samples were also analyzed for ERK and SRF binding: as previously reported, SRF was found to interact with GST-Elk-1 (1-205), where the B domain is found, and ERK/MAPK was found to interact with GST-Elk-1 (205-329), where D docking domain is present, as well as GST-Elk-1 (349-428), where the ERK binding FxF motif is found (Figure 2(b)).

This region encompassing amino acids 1–205 of Elk-1 harbors the DNA binding ETS domain (red box in Figure 2(c)) and was found to contain several binding consensus sites for Aur-A/Aur-B (red underlines, Figure 2(c)), for Plk1 (blue underlines, Figure 2(c)), and for Cdk1 (green underlines, Figure 2(c)), and this predicted binding consensus for mitotic kinases was either overlapping with or in the vicinity of phosphorylation motifs of these kinases (red font for AurA/B, blue font for Plk1, and green font for Cdk1, Figure 2(c)).

### 3.3. *In Vitro* Phosphorylation of Predicted Motifs by Mitotic Kinases

**Figure 3:**
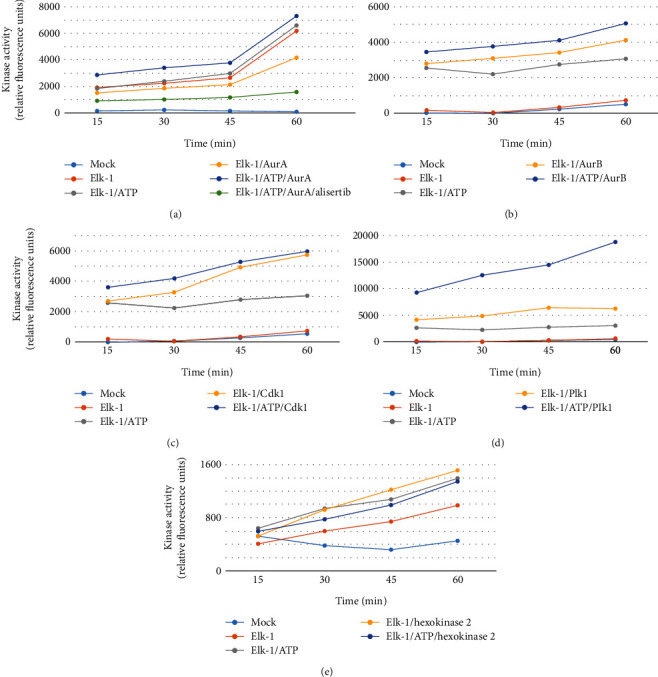
*In vitro* kinase assay for Elk-1 phosphorylation by (a) Aurora-A (AurA), (b) Aurora-B (AurB), (c) Cdk1, (d) Plk1, and (e) Hexokinase 2 kinases. Recombinant Elk-1 protein was incubated with indicated kinases in the presence or absence of ATP, and phosphorylation was monitored by accumulation of ADP at 15, 30, 45, and 60 min time points as determined by increase in fluorescence (i.e., kinase activity was reported as relative fluorescence units). Reaction tube containing only reaction buffer was used as mock.

To confirm whether these predicted motifs were indeed phosphorylated by the predicted kinases, we have carried out *in vitro* kinase assays, incubating recombinant Elk-1 protein with each mitotic kinase separately, and monitored the reaction at 15, 30, 45, and 60?min (Figure 3 for 0.5 ug kinase; see Supplemental File 2 for results with 0.2 ug kinase). Very little or no activity was observed using mock control, recombinant Elk-1 protein alone, or Elk-1 protein with only ATP in any of the reactions with low kinase amount (Supplemental File 2), while in high kinase amount, no or only basal level kinase activity was observed in mock and Elk-1 reactions in almost all reactions (Figure 3). It should be noted that Aur-A, Aur-B, and Cdk1 are sold as active kinases, while no such information is available for other kinases used in assays. When Elk-1 was incubated by itself, in the presence of only ATP, or with 0.5 *μ*g Aurora-A kinase, increased kinase activity was observed over time; however, this activity was decreased to nearly baseline level when Aurora-A inhibitor alisertib was used (Figure 3(a)). There was an increase in kinase activity when Aurora-B was incubated with the recombinant Elk-1 protein, which was further enhanced in the presence of ATP in a time-dependent manner; however, recombinant Elk-1 incubated in the presence of only ATP also resulted in a lower but nonetheless similar kinase activity, which was higher than either mock or Elk-1 only reactions (Figure 3(b)). It should be noted, however, that any potential mitosis-specific posttranslational modifications of Elk-1 have not been represented in this *in vitro* assay.

A more pronounced kinase activation profile was observed with Cdk1 (Figure 3(c)) and Plk1 (Figure 3(d)). Incubation of recombinant Elk-1 with 0.5 *μ*g active Cdk1 kinase either in the presence or absence of ATP resulted in high kinase activity in a time-dependent manner, while mock reaction or reaction with only Elk-1 or Elk-1 with ATP did not yield as high kinase activity as with Cdk1 (Figure 3(c)). The highest kinase activity, however, was observed when Elk-1 was incubated with 0.5 *μ*g Plk1 kinase and ATP as compared to mock, Elk-1, Elk-1 and ATP, or Elk-1 and Plk1 reactions (Figure 3(d)). Custom phosphospecific antibodies showed that endogenous Elk-1 protein was indeed phosphorylated at these predicted residues (Suppl Figure 1). As a negative control, kinase reaction with recombinant Elk-1 protein was performed using hexokinase2, which is known to phosphorylate glucose and result in glucose-6-phosphate; hence, no significant protein phosphorylation was expected. Indeed, when 0.5 *μ*g hexokinase 2 was incubated with Elk-1 in the presence or absence of ATP, baseline activity was observed (Figure 3(e)).

**Figure 4:**
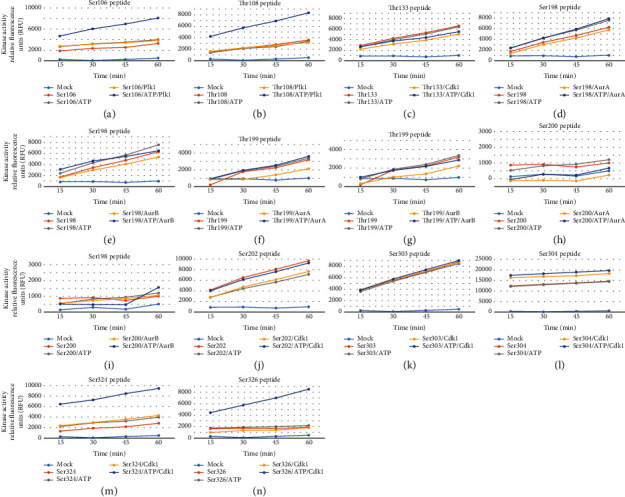
*In vitro* kinase assay for phosphorylation of various Elk-1 peptides by AurA, AurB, Plk1, and Cdk1 kinases. Unmodified peptides (for sequences see Suppl Table 4) were incubated with indicated kinases in the presence or absence of ATP, and phosphorylation was monitored by accumulation of ADP at 15, 30, 45, and 60 min time points. Reaction tube containing only reaction buffer was used as mock, and kinase activity was reported as relative fluorescence units: (a) phosphorylation of Ser106 peptide witth Plk1, (b) phosphorylation of Thr108 peptide with Plk1, (c) phosphorylation of Thr133 peptide with Cdk1, (d) phosphorylation of Ser198 peptide with AurA, (e) phosphorylation of Ser198 peptide with AurB, (f) phosphorylation of Thr199 peptide with AurA, (g) phosphorylation of Thr199 peptide with AurB, (h) phosphorylation of Ser200 peptide with AurA, (i) phosphorylation of Ser200 peptide with AurB, (j) phosphorylation of Ser202 peptide with Cdk1, (k) phosphorylation of Ser303 peptide with Cdk1, (l) phosphorylation of Ser304 peptide with Cdk1, (m) phosphorylation of Ser324 peptide with Cdk1, and (n) phosphorylation of Ser326 peptide with Cdk1. Reaction tube containing only reaction buffer was used as mock.

These results do not specifically address whether specific motifs predicted in this study can indeed be targeted by their putative corresponding kinases. To that end, *in vitro* kinase assays using various unmodified Elk-1 peptides (see Suppl Table 4 for peptide sequences; also see Suppl File 2 for *in vitro* kinase assay results with lower kinase amount) showed that both serine 106 and threonine 108 residue on Elk-1 was phosphorylated by active Plk1 kinase (Figures 4(a) and 4(b)). At lower kinase amounts, threonine 199 and serine 200 residues were found to be phosphorylated by Aur-A, serine 199 was phosphorylated by Aur-B, and serine 202, serine 303, and serine 324 residues were phosphorylated by Cdk1 *in vitro*, albeit with different efficiencies, at low kinase amounts (Suppl File 2). However, at higher kinase amounts, phosphorylation of threonine 133 by Cdk1 (Figure 4(c)), serine 198 by Aur-A (Figure 4(d)) or Aur-B (Figure 4(e)), threonine 199 by Aur-A (Figure 4(f)) or Aur-B (Figure 4(g)), and serine 200 by Aur-A (Figure 4(h)) or Aur-B (Figure 4(i)) appeared to be nonspecific.

Incubation of serine 202, serine 303, or serine 304 peptides with Cdk1 also resulted in nonspecific activation in all reaction conditions (Figures 4(j)–4(l), respectively). However, incubation of serine 324 or serine 326 peptides with Cdk1 resulted in high level of kinase activity specifically in the presence of kinase and ATP (Figures 4(m) and 4(n), respectively).

### 3.4. Protein-Protein Interaction Network of Elk-1 in Mitosis

**Figure 5:**
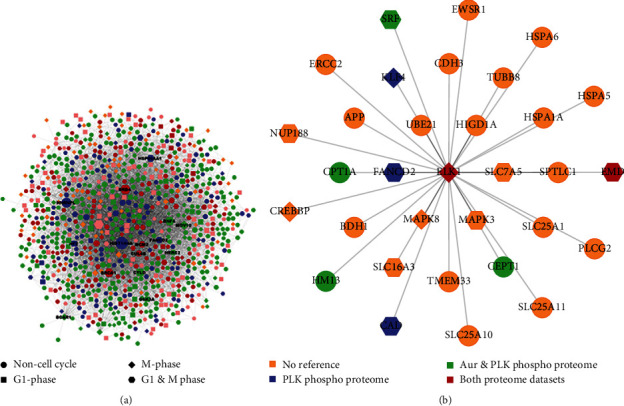
(a) Protein-protein interaction network resulted by integration of human whole PPI network with Elk-1 overexpression microarray in SH-SY5Y cells from KeyPathwayMiner algorithm. (b) Elk-1 and interaction partners in the PPI network. Color codes represent which phosphoproteome dataset of each protein is found in (filtered by mitotic phosphoproteome, Dephoure et al. [23]; only M and G1/M phase proteins are shown in figure for clarity); shape of the nodes represents the cell cycle phase each protein is associated with (filtered with Plk phosphoproteome, Grosstessner-Hain et al. [21], or Aur/Plk phosphoproteome, Kettenbach et al. [22]).

In order to address the implications of phosphorylation of Elk-1 by mitotic kinases, we have next analyzed mitotic interaction network for Elk-1. The protein-protein interaction (PPI) network of Elk-1 in mitotic kinase phosphoproteomes, we have initially integrated human whole PPI network with our previously reported microarray results from SH-SY5Y cells overexpressing constitutively active Elk-1VP16 fusion protein (Figure 5(a); Suppl. Table 2) [26]. The resulting Elk-1-specific PPI network was further filtered with the mitotic phosphoproteomes, which include proteins in G1 phase, M phase, G1&M phase, and noncell cycle proteins, and with the two phosphoproteome sets reported previously for Aurora kinase and Aurora/Plk kinases (Figure 5) [21, 22]. This filtered PPI network included a total of 4292 nodes and 32,851 edges (see Supplemental Table 2); the PPI subnetwork that consists only of G1, M, and G1&M proteins included 945 nodes and 6222 edges (Figure 5(a)).

The direct partners of Elk-1 within this network contained a total of 32 nodes and 31 edges (Figure 4(b)); of those, direct interaction partner for Elk-1was found in both Plk phosphoproteome, and Aur/Plk phosphoproteome (Figure 5(b), red color) was emerin, which is encoded by the EMD gene and is a nuclear membrane protein involved in nuclear actin stabilization and proper nuclear organization; it was further shown to associate with mitotic spindles and centrosomes during mitosis [29].

Elk-1 interaction partners found specifically in Aur/Plk phosphoproteome (Figure 5(b), green color) include its transcriptional partner, SRF, known to coordinate actin cytoskeleton and contribute to mitotic spindle orientation and asymmetric cell division [30–32]. In addition, other Elk-1-interacting proteins include CPT1A, carnitine O-palmitoyltransferase, a mitochondrial membrane enzyme and plays a role in fatty acid oxidation, which is known to promoter metastasis and proliferation in cancer cells [33]; HM13, which catalyzes proteolysis of signal peptides in the ER and was found to contribute to tumor progression [34, 35]; and choline/ethanolamine phosphotransferase CEPT1, which is involved in the synthesis of choline-containing phospholipids, which is reported to be upregulated in certain types of cancer [36].

Elk-1 interaction partners in Plk phosphoproteome (blue color) filtering include KLF4, a regulator of the G1/S checkpoint [37, 38], which was shown to regulate embryonic stem cell self-renewal and a critical regulator of iPSC generation [39]; CAD protein, a protein regulated by MAPK and involved in pyrimidine nucleotide synthesis and shown to regulate a critical rate-limiting step that presents a target for glioblastoma therapy [40]; and FANCD2, which is involved in chromosome stability and repair of DNA double-strand breaks, localizes to specific sites on mitotic chromosomes where replication fork has stalled and acts as a primary supporter of mitotic DNA synthesis in cancers [41].

Other mitotic phase-dependent partners of Elk-1 that are not referenced in either Plk or Aur/Plk phosphoproteomes include G1&M partners nucleoporin NUP188 and MAPK3 (ERK1), M phase partners histone acetyltransferase CREBBP and MAPK8 (JNK), and noncell cycle partners RNA binding protein EWSR1, cadherin CDH3, tubulin TUBB8, hypoxia-inducible protein HIGD1A, amyloid precursor protein APP, ubiquitin-conjugating enzyme UBE21, and mitochondrial proteins SLC25A1 (mitochondrial citrate transporter), SLC25A11 (mitochondrial malate carrier), and SLC25A10 (mitochondrial dicarboxylate carrier). Nucleoporin NUP188 has been shown to localize to spindle poles during mitosis and regulate centriole duplication as well as chromosome segregation [42, 43]. EWS RNA binding protein 1, or EWSR1, is a multifunctional protein; it was found to be spindle-associated and to regulate microtubule acetylation in mitotic spindles in a cell cycle-dependent manner [44]. Interestingly, EWSR1-ETS fusions are quite common in Ewing sarcomas [45]. TUBB8, on the other hand, was found to be essential for oogenesis and was shown to be important in spindle assembly [46]. Mitochondria are highly dynamic organelles, and it has recently been shown that the cell cycle-dependent centromeric protein CENP-F was recruited to mitochondria during mitosis to ensure redistribution upon cell division [47].

## 4. Discussion

Mitotic kinase Aurora-A is overexpressed in glioma cells, and its expression is reported to be correlated with patient outcome [48]; furthermore, Plk1 activity was found to be elevated in CD133+ glioblastoma multiforme cells [49]. Mitotic kinases have therefore been a focus of attention for designing small molecule inhibitors of cancer: mammalian Aurora-A kinase received particular attention for cancer therapeutics not only through its mitotic functions but also nonmitotic functions such as its role in epithelial-mesenchymal transition [50].

We had previously shown that Elk-1 interacted with axonal microtubules and relocated to the cell nucleus upon stimulation and that Elk-1-dynein interaction was independent of serum stimulation [10, 11]; we have shown that in the presence of Aur-A, Cdk1, Plk1, and dynein inhibitors, P-S383-Elk-1 species fails to localize to the spindle poles at the onset of mitosis and relocalize to midzone and midbody as mitosis progresses but instead is colocalized with DNA in dot-like structures [11, 12] (data not shown).

It should be noted that while phosphorylation sites for mitotic kinases showed potential phosphorylation in increasing lysate amounts (Figure 3), interaction with mitotic kinases was observed only on GST-Elk (1-205) region (Figure 2), much like the ERK binding domain on Elk-1 (the D domain, aa 312-328) and the ERK/MAPK phosphorylation motifs (Ser383 and Ser389). In this study, we present not only interaction of Elk-1 with Aurora-A but also for the first time its interaction with other mitotic kinases Aurora-A, Plk1, and Cdk1 and identify the interaction domain to be within the first 205 amino acids of Elk-1 protein. Further studies should be carried out to narrow down the specific domains of interactions for individual mitotic kinases.

Fluorescence-based kinase assays show that the predicted phosphorylation motifs were indeed phosphorylated *in vitro*, although the phosphorylation *in vivo* needs to be studied further in detail. Additionally, while the binding domain for all these mitotic kinases is found within the N-terminal half of Elk-1 and the corresponding phosphorylation motifs are elsewhere on the protein, it will be interesting to investigate whether phosphorylation of Elk-1 by one mitotic kinase can affect its binding to another mitotic kinase.

Protein-protein interaction network of Elk-1 filtered by mitotic phosphoproteome or mitotic kinase phosphoproteomes also identified potential partners for Elk-1 during mitosis, such as EMD, KLF4, and NUP188, in addition to previously known partners such as SRF, MAPK3, and MAPK8, further indicating a potential role of Elk-1 in mitosis. Preliminary FACS analyses have indeed shown that when phosphomutants of Elk-1 were overexpressed in cell lines, mitotic profile of cells released from nocodazole arrest was found to be altered (Suppl. Figure 2 and Suppl. Tables 1 and 3). Since many of the mitosis-dependent interaction partners for Elk-1 have previously been reported to play a role in mitosis, such as spindle assembly or chromosome segregation, it will be interesting to identify whether mitotic kinase phosphorylation of Elk-1 can affect interaction with these proteins and whether such an alteration can affect mitosis progression in cells.

## Data Availability

The authors provide only a subset of data in the supplemental files for space concerns; all other data are available upon request.

## Abbreviations

ADP: Adenosine diphosphate
APP: Amyloid precursor protein
ATP: Adenosine triphosphate
Aur-A: Aurora A
Aur-B: Aurora B
Cdk1: Cyclin-dependent kinase-1
CENP: Centromere protein
CEPT: Choline/ethanolamine phosphotransferase
CPT1A: Carnitine O-palmitoyltransferase 1A
CST: Cell signaling technologies
EHNA: Erythro-9-(2-hydroxy-3-nonyl) adenine
Elk-1: Ets-like protein 1
ERK: Extracellular regulated kinase
ETS: E-twenty-six/erythroblast/E26-transformation-specific
FACS: Fluorescence-activated cell sorting
FBS: Fetal bovine serum
FSC: Forward scatter
GST: Glutathione S-transferase
HIGD1A: HIG1 domain family member 1A
HRP: Horseradish peroxidase
MAPK: Mitogen-activated protein kinase
NLS: Nuclear localization signal
PBS: Phosphate buffered saline
PEI: Polyethylenimine
PI: Propidium iodide
PPI: Protein-protein interaction
PI3K: Phosphatidyl inositide-3 kinase
PKC: PI3K/Akt and protein kinase C
Plk1: Polo-like kinase-1
PPI: Protein-protein interaction
PKC: Protein kinase C
SSC: Side scatter
SRF: Serum response factor
SUMO: Small ubiquitin-like modifier
TAD: Transactivation domain
TCF: Ternary complex family
UBE: Ubiquitin-conjugating enzyme.

## Supplementary Materials

Supplementary Materials Supplemental Table 1: the adjusted percentages of cell fractions in different stages of the cell cycle. Supplemental Table 2: protein-protein interaction network resulted from KeyPathwayMiner algorithm. Supplemental Table 3: sequences of mutagenic forward primers and nonmutagenic reverse primers used in site-directed mutagenesis. Supplemental Table 4: modified phosphopeptides against which antibodies were raised by GenScript and their unmodified counterparts used as negative control in dot blot analyses. Supplemental Figure 1: phosphorylation analysis of Elk-1. Supplemental Figure 2: the effect of Elk-1 phosphorylation mutations on cell cycle profiles. Supplemental File 1: original representative Western blot images (slides 1-31). Supplemental File 2: kinase assay recipe and kinase assays with lower kinase amount.
