# Supplementary material for: Mitotic Kinases Aurora-A, Plk1, and Cdk1 Interact with Elk-1 Transcription Factor through the N-Terminal Domain
Source: Int J Cell Biol. 2024 Apr 30;2024:6798897. doi: 10.1155/2024/6798897 (PMC11074830; doi:10.1155/2024/6798897)
Supplement: Supplementary Materials — Supplemental Table 1: the adjusted percentages of cell fractions in different stages of the cell cycle. Supplemental Table 2: protein-protein interaction network resulted from KeyPathwayMiner algorithm. Supplemental Table 3: sequences of mutagenic forward primers and nonmutagenic reverse primers used in site-directed mutagenesis. Supplemental Table 4: modified phosphopeptides against which antibodies were raised by GenScript and their unmodified counterparts used as negative control in dot blot analyses. Supplemental Figure 1: phosphorylation analysis of Elk-1. Supplemental Figure 2: the effect of Elk-1 phosphorylation mutations on cell cycle profiles. Supplemental File 1: original representative Western blot images (slides 1-31). Supplemental File 2: kinase assay recipe and kinase assays with lower kinase amount. [file 6798897.f1.zip › Supplementary Materials.docx]

**Supplementary Materials**

1. **Supplemental Files (supplementalfigurelegends-rev-feb2024.pdf)**

**Supplemental Table 1.** The adjusted percentages of cell fractions in different stages of the cell cycle (see Suppl Fig 1)

**Supplemental Table 2.** Protein-Protein interaction network resulted from KeyPathwayMiner algorithm. V1 and V2 column represent the nodes which has physical interaction. The other column indicates the information about node column used in color code in Figure 4. Information of V1 nodes placed in _1 columns and V2 nodes placed in _2 columns.

**Supplemental Table 3.** Sequences of mutagenic forward primers and non-mutagenic reverse primers used in site-directed mutagenesis. Mutagenic nucleotides are shown in boldface and underlined in forward primers.

**Supplemental Table 4.** Modified phospho-peptides against which antibodies were raised by GenScript, and their unmodified counterparts used as negative control in dot blot analyses

**Supplemental Figure 1.** Phosphorylation analysis of Elk-1. Increasing amounts of untransfected U87 glioblastoma cell lysates were analyzed with phospho-specific antibodies (upper panels, block arrows) as well as for total Elk-1 (bottom panel, 62 kDa protein indicated by block arrow) as loading control after stripping and re-probing. To the left of each blot, the phosphorspecific primary antibodies used in Western blots were indicated (P-S106-Elk-1, P-T108-Elk-1, total Elk-1 etc), and the leftmost boldface writings in parantheses indicate the mitotic kinases that are predicted to phosphorylate these motifs.

**Supplemental Figure 2.**  The effect of Elk-1 phosphorylation mutations on cell cycle profiles. SH-SY5Y cells were transfected with empty pCMV-Flag plasmid as well as plasmids expressing wildtype Elk-1 (pCMV-Elk-1) and S106A, T108A, T133A, S198A and T199A phosphomutants of Elk-1 (left panel), or with plasmids encoding S149A, S303A, S304A, S324A, and -S326A phosphomutants of Elk-1 (right panel). 24 hrs later cells were treated with 100 ng/ml nocodazole for 16 hr to arrest cells, followed by release into mitosis. Cells were then collected at the time of release, as well as 30, 60 or 90 min after release, and analyzed by flow cytometry as described in Materials and Methods. Asynchronous cell population was separately studied. The results were analyzed and normalized to 100 % of total cells. Blue bars represent % cells in G1, orange bars represent % cells in S, and grey bars represent % cells in G2 (see Suppl Table 1).

**Supplemental Materials and Methods**

**Plasmids**

The mutation at S383A was inserted into Flag-tagged pCMV-5-Flag-Elk-1 plasmid (kindly provided by Prof. A.D. Sharrocks, UK) by Gene Tailor Mutagenesis Kit (Invitrogen), following manufacturer’s instructions. Briefly, the wild-type plasmid was first methylated and then the whole plasmid was amplified with primers (5’-ATTCACTTCTGGAGCACCCTG**GC**TCCCATTGCG-3 and 5’-CAGGGTGCTCCAGAAGTGAATGCTAGGAGG-3’) carrying the mutation (the mutation-bearing nucleotides are indicated in boldface and underlined). Then, the linear PCR product was transformed into DH5α-T1 strain, positive colonies were selected and verified by sequencing.

**Site-directed mutagenesis of Elk-1**

pCMV-5-Flag-Elk-1 plasmid was used as a template for site-directed mutagenesis of predicted phosphorylation motifs. Target serine (S) or Threonine (T) amino acids were mutated to either alanine (A) or glutamic acid (E) residues by specific primers designed according to NEBaseChanger tool (Suppl Table 3). NEB Q5 site-directed mutagenesis kit (E0554S) was used to mutated target sequences on pCMV-5-Flag-Elk-1 plasmid, as per manufacturer’s instructions, and non-overlapping primers were designed using the manufacturer’s online primer design tool, NEBaseChanger (<https://nebasechanger.neb.com/>).

**Flow cytometry**

3x10^5^ SH-SY5Y cells were seeded onto 6-well plates and transfected with 2 µg of wild-type or mutant Elk-1 plasmids by PEI transfection reagent. Cells were collected by trypsin treatment and treated with 20% paraformaldehyde for fixation for 10 minutes. Cells were then incubated with 0.5% BSA prepared in 0.1%Triton-X-100 solution for both blocking and permeabilization for 5 min at room temperature. Alexa Fluor 488 conjugated anti- Histone H3 pSer10 antibody (Cell Signaling #9708) was diluted in 0.5% BSA solution in a 1:100 dilution ratio and cell pellet were dissolved. The cells and antibody resuspension were incubated in dark for 45 minutes at room temperature. PI staining solution was prepared in 0.1% Triton X-100 in PBS, at final concentrations of 1 µg/ml PI and 20 µg/ml DNase-free RNase A and cells were stained for 30 minutes and were run on flow cytometer at appropriate FSC (Forward Scatter) and SSC (Side Scatter) gates to exclude cell debris and aggregates. % cells in G1 (M1), S (M2) and G2 (M3) were exported as MS Excel file, and the sum of G1+S+G2 were normalized to 100 % for all experimental sets, and data was presented as bar graph.

**Phospho-specific antibody generation**

Immunogenic peptide and phosphopeptide epitope sequences including predicted phosphorylation sites were designed, and phospho-specific antibodies were custom-synthesized by GenScript company against these phospho-modified peptides and selected against unmodified peptides (ELISA reports provided by the company; for peptide sequences: [27] and Suppl Table 4). The phospho-specific antibodies as well as the preimmune serum (all provided by the company) received were initially analysed using phospho-modified and unmodified peptides in dot blot assays (data not shown). The phospho-specific antibodies thus confirmed were then used in Western blot assays. (Kurnaz I, Uyar OA, Yilmaz B. Mitotic kinase phosphorylation epitopes of Elk-1 protein and phosphospecific antibodies against these epitopes; TurkPatent application 2019; reference no 133P28)

***In vitro* protein kinase assay**

Parallel to the kinase assay presented in Figure 3, this fluorescent  *in vitro* kinase reaction was set up with the incubation of unmodified Elk-1 peptides described in Suppl Table 4 with the indicated kinases. 0.1 µg Elk-1 peptides were incubated with either Aurora A (Millipore 14-511), Aurora B (CST #7394), Cdk1/Cyclin B1 (Millipore 14-450) or Plk1 (CST #7728) active kinases at 37°C for 1 hour, as per manufacturer’s instruction (Universal Kinase Assay Kit, abcam ab138879). Then, 20 µl kinase reaction was combined with 20 µl ADP sensor buffer and 10 µl ADP sensor composed of the mixture of ADP sensor I and II. The mixture was incubated in dark for 15 minutes and the fluorescence intensity was measured by spectrophotometry at 540 nm excitation and 590 nm emission (Suppl. Fig 2)

**Supplemental Results**

**Detection of Elk-1 phosphorylation using phospho-specific antibodies**

Upon confirming physical interaction between Elk-1 protein and mitotic kinases, we have studied phosphorylation of predicted motifs by these kinases using custom-generated antibodies against selected predicted motifs, and phospho-specificity was confirmed using phospho-modified and unmodified peptides in dot blots (data not shown). Our results show increasing signal intensities on 10, 20 and 40 μg of non-transfected U87 lysates with P-S106-, P-T133-, P-T199, P-S200, P-S303-, P-S304-, P-S324- and P-S326-Elk-1 antibodies, indicating potential endogenous phosphorylation of these residues, while P-T108- and P-S202-Elk-1 antibodies gave weak signals (Suppl Fig. 1; total Elk-1 antibody was used as loading control).

**The effect of Elk-1 phospho-mutants on mitosis**

So far we have shown that Elk-1 interacts with mitotic kinases in a mitotic stage-dependent manner,and that mitotic kinase inhibitors do indeed affect the mitotic localization of P-S383-Elk-1 species, and phosphorylation analysis had been performed in U87 cells (Fig.4). However to address whether these phosphorylations are directly relevant to the mitotic profile of cells, we have next overexpressed different phosphomutants in a different cell line, in SH-SY5Y cells, and studied their mitotic progression (Suppl Fig.2). When cells transfected with empty pCMV-Flag plasmid is arrested and released into mitosis, the population of cells entering G1 gradually increase, almost displaying the profile of asynchronous cells within 90 min (Suppl Fig.2). Cells transfected with pCMV-Elk-1 eventually enter G1, as do S106A, T108A, T133A, S198A and T199A mutants. Similar is true for S149A-Elk-1 transfected cells in a parallel set of experiments, however S303A, S304A, S324A and S326A mutants appear to have defects in entering mitosis when compared to cells transfected with S149A mutant (Suppl Fig.2; also see Suppl Table 1).

1. **Supplemental File 1.** Original representative Western blot images (slides 1-31).

**(supplementalfile1_rawdata.**pdf)

1. **Supplemental File 2.** Kinase assay recipe and kinase assays with lower kinase amount

**(supplementalfile2-**fluorometric kinase assay.pdf)

**Kinase assay recipe**

**(**PhD thesis: https://tez.yok.gov.tr/UlusalTezMerkezi/tezSorguSonucYeni.jsp; thesis no: 538994**)**

Plk1, Threonine 199 and Serine 200 were incubated with either Aurora A or Aurora B, Threonine 133, Serine 202, 303, 304 and 326 were incubated with Plk1 at 37oC for 1 hour. Then, 20 μl kinase reaction was combined with 20 μl ADP sensor buffer and 10 μl ADP sensor composed of the mixture of ADP sensor I and II. The mixture was incubated in dark for 15 minutes and the fluorescence intensity was measured by spectrophotometry at 540 nm excitation and 590 nm emission.

Thesis Table 4.13 Components of fluorometric kinase assay reaction

| Elk-1 protein | | Elk-1 peptides | |
| --- | --- | --- | --- |
| Component | Final Concentration | Component | Final Concentration |
| Elk-1 | 0.1 μg | peptides | 1 mg/ml |
| Kinases | 0.2 μg | Kinases | 0.2 μg |
| ATP | 200 mM | ATP | 200 mM |
| ADP assay buffer | 50 μl | ADP assay buffer | 25 μl |

**Kinase assays with recombinant Elk-1 protein**

To confirm whether these predicted motifs were indeed phosphorylated by the predicted kinases, we have carried out in vitro kinase assays, incubating Elk-1 protein with each mitotic kinase separately, and monitored the reaction at 15, 30, 45 and 60 min. Very little or no activity was observed using mock control, recombinant Elk-1 protein alone, or Elk-1 protein with only ATP in any of the reactions. There was an increase in kinase activity when Aurora-A or Aurora-B was added to the recombinant Elk-1 protein, which was further enhanced in the presence of ATP and increased with incubation time (Suppl File 2, Fig.A/slide 3 and B/slide 4). A similar profile was observed with Cdk1 (Suppl File 2, Fig.C/slide 5) and Plk1 (Suppl File 2, Fig.D/slide 6), although the level of activation was significantly lower than those for either Aurora-A or Aurora-B (compare to Suppl File 2, Fig.A and D). Custom phospho-specific antibodies showed that endogenous Elk-1 protein was indeed phosphorylated at these predicted residues (Suppl Fig 1).

**Kinase assays with Elk-1 peptides**

Meanwhile, in vitro kinase assays using various unmodified Elk-1 peptides (see Suppl Table 4 for peptide sequences) showed that Threonine 108 residue on Elk-1 was phosphorylated by active Plk1 kinase, Threonine 199 and Serine 200 residues were phosphorylated by Aur-A, Serine 199 was phosphorylated by Aur-B, and Serine 202, Serine 303 and Serine 324 residues were phosphorylated by Cdk1 in vitro, albeit with different efficiencies (Suppl File 2, Fig.on slide 7). Briefly, when unmodified Ser106 peptide was incubated in the presence of active Plk1 kinase and ATP, no significantly different phosphorylation was quantified when compared to Ser106 peptide alone (Suppl File 2, Fig.A, slide 9), while Thr108 peptide was phosphorylated in a time-dependent manner when incubated in the presence of active Plk1 kinase and ATP (Suppl File 2, Fig.B/slide 10). On the other hand, Thr133 peptide showed non-specific phosphorylation, since Thr133 peptide alone showed higher phosphorylation than Thr133 peptide with ATP (Suppl File 2, Fig.C/slide 11). Ser198 peptide by itself showed high phosphorylation as measured by kinase activity, which did not significantly increase upon incubation with ATP or AurA, AurB or Plk1 kinases; in fact, AurB incubation resulted in even lower phosphorylation than peptide alone (Suppl File 2, Fig.D/slide 12). On the other hand, Thr199 (Suppl File 2, Fig.E/slide 13) and Ser200 (Suppl File 2, Fig.F/slide 14) peptides both showed time-dependent increase in phosphorylation in the presence of ATP and either AurA or AurB kinases. Similarly, incubation of Ser202 (Suppl File 2, Fig.G/slide 15) and Ser303 (Suppl File 2, Fig.H/slide 16) peptides with both ATP and Cdk1 resulted in a timedependent increase in phosphorylation in vitro. However, Ser304 peptide showed time-dependent phosphorylation only with AurA incubation but not Cdk1 (Suppl File 2, Fig.I/slide 17). Ser324 peptide assays are presented in slide 18. Finally, Ser326 peptide was not significantly phosphorylated by either Cdk1 or Plk1 in vitro (Suppl File 2, Fig.J/slide 19)
