# Supplementary figures and images for: Mitotic Kinases Aurora-A, Plk1, and Cdk1 Interact with Elk-1 Transcription Factor through the N-Terminal Domain
Source: Int J Cell Biol. 2024 Apr 30;2024:6798897. doi: 10.1155/2024/6798897 (PMC11074830; doi:10.1155/2024/6798897)

lysate

10  $\mu$ g      20  $\mu$ g      40  $\mu$ g

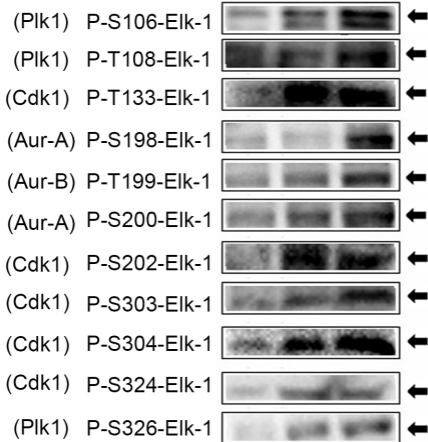

total Elk-1

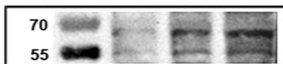

← 62 kDa

Supplement: Supplementary Materials — Supplemental Table 1: the adjusted percentages of cell fractions in different stages of the cell cycle. Supplemental Table 2: protein-protein interaction network resulted from KeyPathwayMiner algorithm. Supplemental Table 3: sequences of mutagenic forward primers and nonmutagenic reverse primers used in site-directed mutagenesis. Supplemental Table 4: modified phosphopeptides against which antibodies were raised by GenScript and their unmodified counterparts used as negative control in dot blot analyses. Supplemental Figure 1: phosphorylation analysis of Elk-1. Supplemental Figure 2: the effect of Elk-1 phosphorylation mutations on cell cycle profiles. Supplemental File 1: original representative Western blot images (slides 1-31). Supplemental File 2: kinase assay recipe and kinase assays with lower kinase amount. [file 6798897.f1.zip › SuppFig1.pdf]

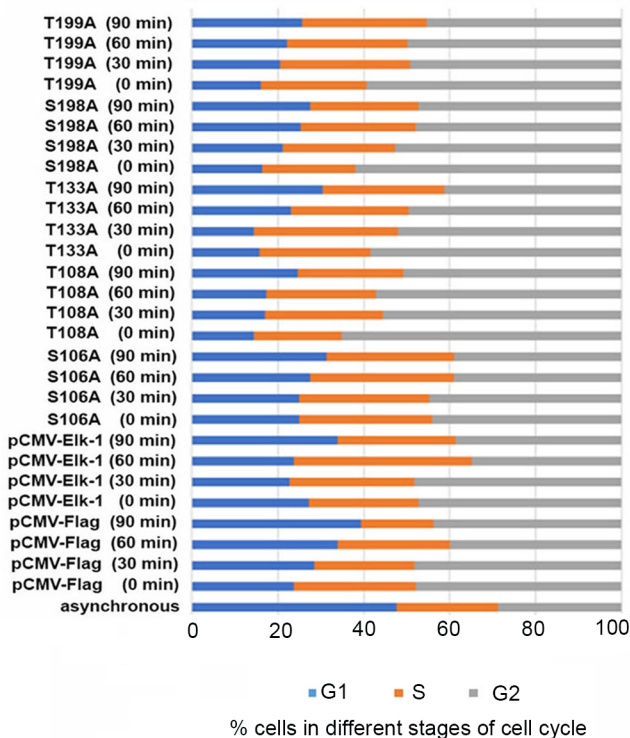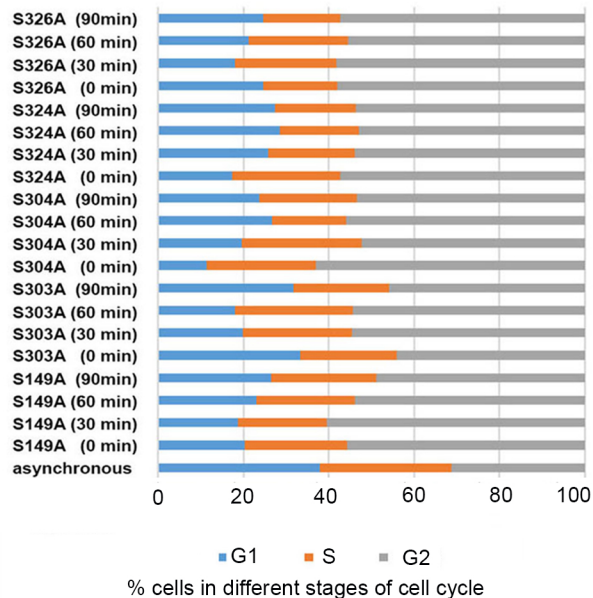

Supplement: Supplementary Materials — Supplemental Table 1: the adjusted percentages of cell fractions in different stages of the cell cycle. Supplemental Table 2: protein-protein interaction network resulted from KeyPathwayMiner algorithm. Supplemental Table 3: sequences of mutagenic forward primers and nonmutagenic reverse primers used in site-directed mutagenesis. Supplemental Table 4: modified phosphopeptides against which antibodies were raised by GenScript and their unmodified counterparts used as negative control in dot blot analyses. Supplemental Figure 1: phosphorylation analysis of Elk-1. Supplemental Figure 2: the effect of Elk-1 phosphorylation mutations on cell cycle profiles. Supplemental File 1: original representative Western blot images (slides 1-31). Supplemental File 2: kinase assay recipe and kinase assays with lower kinase amount. [file 6798897.f1.zip › SupplFig2.pdf]
